# Effect of Foot Hyperpronation on Lumbar Lordosis and Thoracic Kyphosis in Standing Position Using 3-Dimensional Ultrasound-Based Motion Analysis System

**DOI:** 10.5539/gjhs.v6n5p254

**Published:** 2014-06-16

**Authors:** Khatere Farokhmanesh, Toraj Shirzadian, Mohammad Mahboubi, Mina Neyakan Shahri

**Affiliations:** 1 Orthotics and Prosthetics, Kermanshah University of Medical Science’s, Kermanshah, Iran; 2 Kermanshah University of Medical Science’s, Kermanshah, Iran; 3 Health Services Administration, Kermanshah University of Medical Science’s, Kermanshah, Iran; 4 Ahvaz Jundishapur University of Medical Sciences, Abadan College of Medical Sciences and Health Services, Ahvaz, Iran; 5 Kermanshah University of Medical Science’s, Kermanshah, Iran

**Keywords:** hyperpronation, foot, lumbar lordosis, thoracic kyphosis, wedge, motion analysis system

## Abstract

Based on clinical observations, foot hyperpronation is very common. Excessive pronation (hyperpronation) can cause malalignment of the lower extremities. This most often leads to functional and structural deficits. The aim of this study was to assess the effect of foot hyperpronation on lumbar lordosis and thoracic kyphosis. Thirty five healthy subjects (age range, 18030 years) were asked to stand on 4 positions including a flat surface (normal position) and on wedges angled at 10, 15, and 20 degrees. Sampling was done using simple random sampling. Measurements were made by a motion analysis system. For data analysis, the SPSS software (ver. 18) using paired t-test and repeated measures analysis of variance (ANOVA) was applied.

The eversion created by the wedges caused a significant increase in lumbar lordosis and thoracic kyphosis. The most significant change occurred between two consecutive positions of flat surface and the first wedge. The t-test for repeated measures showed a high correlation between each two consecutive positions.

The results showed that with increased bilateral foot pronation, lumbar lordosis and thoracic kyphosis increased as well. In fact, each of these results is a compensation phenomenon. Further studies are required to determine long-term results of excessive foot pronation and its probable effect on damage progression.

## 1. Introduction

Foot alignment, as the most distal part of the lower extremity kinematic chain as well as providing support to maintain the body’s balance, has an important role in standing and walking (Ledoux & Hillstrom, 2002). The normal biomechanics of the foot can be disrupted as a result of abnormal function of the subtalar joint. In a closed kinematic chain, subtalar joint pronation is associated with adduction and plantar flexion of the talus as well as calcaneal eversion. Unilateral or bilateral calcaneal eversion can result in pathologic conditions in the spinal column (Khamis & Yizhar, 2007). Studies have demonstrated that in a closed kinematic chain in healthy subjects, foot hyperpronation causes internal rotation of the shin and thigh and consequently results in increased pelvic anteversion (Khamis & Yizhar, 2007; Pinto et al., 2008). This ends in more kinematic changes during walking, running, climbing and descending a ladder, activities in which more forces are made to the body. Pelvic posture, acting as an intermediate between the spinal column and the lower extremities, is the main key to suitable postural alignment (Poussa et al., 2005). Pelvic posture has significant association with lumbar vertebral posture (Levine & Whittle, 1996). When the body’s center of gravity is deviated from its ideal alignment, postural compensation strategies are employed to achieve a stable posture. This helps the body’s center of gravity return to a stable condition within the base of support’s span (Shumway-Cook & Woollacott, 2001). Since postural changes of the lower extremities can lead to postural changes in pelvic girdle and enhance the risk of low back pain progress, foot alignment should be considered as an important and effective factor (Rothbart & Estabrook, 1988). The spinal column is basically an unstable structure (Panjabi, 1992). Instability of the spinal column is a complex issue which is not understood completely yet. Hence, there is need for more biomechanical and physiologic studies in this regard. The curvatures in the vertebral column cause flexibility, mobility, and stability and if any of these curvatures is affected pathologically, the function of the spinal column will be disturbed (Fon, Pitt, & Thies, 1980). In previous studies, the relationship between foot hyperpronation and the lower extremity alignment and pelvic position has been investigated. Based on clinical observations, foot hyperpronation is very common and may cause malalignment of the lower extremities. This can cause structural and functional deficits in standing and walking. Understanding the biomechanical structure of each part of the body is important in order to prevent and effective treatment of each part of the musculoskeletal system (Abboud, 2002). There is no evidence to suggest relationship between foot hyperpronation and spine alignment. However, since the body parts acts like a chain and each part affects another part, there is the possibility that change in foot alignment affects the spine and when the curvatures of the vertebral column change, instability occurs in the spine (Sinaki & Mikkelsen, 1984). This can also lead to imbalance and structural abnormalities in patient (Saunders & Ryan, 2004). Considering the importance of foot alignment and its effect on the lower extremities, pelvic girdle, and spine in a closed kinematic chain, the objective of this study is to assess the immediate effect of foot hyperpronation on lumbar lordosis and thoracic kyphosis.

## 2. Materials and Methods

This was a quasi-experimental interventional study. The sampling method was simple random sampling. The sample size was calculated using the following formula:


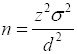


In 2012, 35 healthy men with age range of 18-30 years were selected. Firstly, all participants completed the informed consent form as well as questionnaire of exclusion criteria, selection, data documentation, and observations. Exclusion criteria were inflammatory disease, muscular atrophy, tumor, taking medicines affecting the central nervous system function, spinal column disorders such as scoliosis, structural abnormalities in the lower extremities such as flatfoot, lower limbs length discrepancy more than 5 mm, and acute or chronic low back pain.

Firstly, anthropometric characteristics including age, height, and weight of the subjects were recorded using measuring tape and digital weight scale machine (Table 1). Then, an ultrasonic motion analysis system (Zebris®, Germany) which included a receiver device with high accuracy and an ultrasound pointer stick to define reference points on the skeleton and precise display on the computer screen was used to document posture and shape of the spine and measure lumbar lordosis and thoracic kyphosis. This system is more convenient to use compared to Vicocn systems, radiography, and goniometer. Test-retest reliability of this system ranges from 0.997 to 1 and its validity is between 0.988 and 1 (Prange et al., 2002).

**Table 1:** Central tendency and dispersion indices of the studied variables

	Mean	Standard deviation	Minimum	Maximum
Age (year)	22.8	2.89	18	28
Weight (Kg)	78	7.77	61.8	93.5
Height (cm)	177	4.98	168	187
Body mass index	24.8	2.73	19.8	31.2

**Figure 1:**
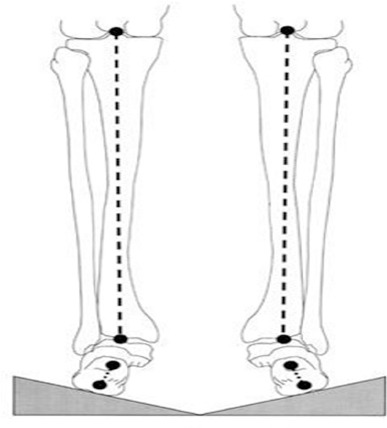
Wooden wedges

Before starting the test, the measuring device was calibrated to define its position related to earth. The study subject stood in a relaxed position in an 80 cm distance from sensor device in two positions, one position was on a flat surface and the other position was standing on wedges with different angles (58 cm × 58 cm). The wooden wedges had two similar slopes at 10, 15, and 20 degrees, were tilted inward and were connected to each other at the center in the lowest point (Figure 1). Reference marker was attached to the subject’s body using a Velcro strap to minimize body positional fluctuations during measurement by ultrasound pointer stick. The position of the examiner was preferably on the opposite side of the reference marker. After determining anatomical points of the subject’s body by the examiner with pressure applied to the pointer stick, the points of shoulder acromion (left and right) and anterior superior as well as posterior superior iliac spine projections (left and right) were entered to the system. It lasted one second for each point entered to be displayed on the screen and then there was a short audio signal. In this study C7 vertebral spinous process was considered as the starting point of the curvature and S3 vertebral spinous process was considered as the terminal point of the curvature. After determining the required bony points, the point of the pointer stick was moved on the spinous processes of the vertebrae and finally the shape of that part of the spine was scanned and displayed on the computer screen (Prange, 2002; Byfield & Kinsinger, 2002; Youdas, Suman, & Garrett, 1995). These measurements were made by two examiners and each measuring method was repeated for three times. The gathered data were analyzed using the SPSS software (ver. 21.0). To determine the normal distribution of the variables, the Kolmogorov-Smirnov test was used. To compare the variables, the paired t-test was applied. To analyze the effect of wedges by the two examiners, repeated measures analysis of variance (ANOVA) was used. This project was approved by the Ethics Committee of Iran University of Medical Sciences and all ethical issues were respected in all stages of the study.

## 3. Results

The Kolmogorov-Smirnov test showed that the variables had a normal distribution. Hence, parametric tests were used to analyze the data. To evaluate the effect of each wedges by the two examiners, the repeated measures ANOVA and finally since the comparisons were pairwise, to compare each variable at 4 different positions assessed by the examiners, the paired t-test was applied (Table 2). To determine the difference among the four variables, we made use of pairwise comparisons of the variables (Bonferroni correction). The significance level of the Bonferroni test for six pairwise comparisons and considering alpha of 5% was 0.0083.

**Table 2:** Comparison of variables at four standing positions on flat surface, and on a 10º, 15º, and 20º wedge by two examiners (mean ± standard deviation, SD)

Variable	Examiner	Flat surface (mean ± SD)	10º wedge (mean ± SD)	15º wedge (mean ± SD)	20º wedge (mean ± SD)
Lumbar lordosis	Examiner 1	30.2 (±5.21)	32.8 (±5.45)	34.5 (±5.50)	36.8 (±5.56)
	Examiner 2	30.5 (±5.75)	32.7 (±5.31)	34.7 (±5.65)	36.9 (±5.78)
Thoracic kyphosis	Examiner 1	31.2 (±5.32)	31.9 (±5.19)	33.4 (±5.78)	34.8 (±5.52)
	Examiner 2	31.0 (±5.00)	32.1 (±5.15)	33.4 (±5.77)	34.8 (±5.66)

α’ = 0.05/6= 0.0083

Therefore, whenever the significance level of pairwise comparisons was lower than 0.0083, it was considered significant, otherwise it was non-significant. The paired t-test results showed that there were significant differences between all modes for lumbar lordosis and thoracic kyphosis. P value for all variables was 0.000 (P< 0.0083); Table 3. Finally, a positive correlation for the examiners and lumbar lordosis as well as thoracic kyphosis was achieved by increasing the angle of the wedge.

**Table 3:** Comparison of lumbar lordosis and thoracic kyphosis in standing positions on flat surface and on 10º, 15º, and 20º wedges

	T test value, lordosis	Significance level, lordosis	T test value, kyphosis	Significance level, kyphosis
Flat surface and 10º wedge	- 5.962	0.000*	- 2.448	0.000*
Flat surface and 15 º wedge	- 14.387	0.000*	- 6.198	0.000*
Flat surface and 20 º wedge	- 23.346	0.000*	- 10.315	0.000*
10º and 15 º wedges	- 9.993	0.000*	- 6.032	0.000*
10º and 20º wedges	- 17.959	0.000*	- 8.365	0.000*
15º and 20º wedge	- 14.829	0.000*	- 7.646	0.000*

* Significant

**Figure 2:**
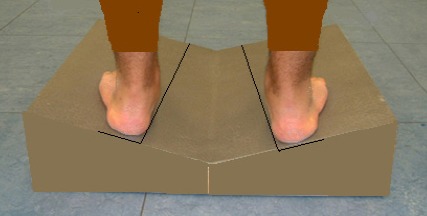
Wooden wedges

Simultaneous mean, first quartile, third quartile, minimum, and maximum values of lumbar lordosis and thoracic kyphosis were compared to each other at 4 measuring positions (the lines at the middle of rectangular which are parallel to length axis are median, two lines which are respectively below and above each rectangular are minimum and maximum, and lower and higher widths are respectively the first and third quartiles). It is obvious from this figure that with increasing angle of the wedge, the values of minimum, the first quartile, median, the third quartile and the maximum all increased (Figure 2).

## 4. Discussion

In some previous studies, wooden wedges with angles of 10º, 15º, and 20º have been used to create foot hyperpronation. These wedges change the alignment of the subtalar joint in a way that with increasing the slope of the wedges, eversion increases significantly. Therefore, in this study similar wedges with the mentioned angles were used (Khamis & Yizhar, 2007; Pinto, 2008).

To analyze lumbar lordosis and in order to assess difference between the variables and also between the two examiners, repeated measures ANOVA was used. Significance value of this test for the variables was P=0.000 and for the examiners it was P= 0.937. These demonstrate that there was difference between the variables at 4 evaluated positions, but there was no difference between the examiners. Correlation for the examiner 1 and lumbar lordosis at different standing positions between flat surface and 10º wedge, flat surface and 15º wedge, flat surface and 20º wedge were respectively 0.807, 0.852, 0.863. Correlation for the examiner 2 and lumbar lordosis at different standing positions between flat surface and 10º wedge, flat surface and 15º wedge, flat surface and 20º wedge were respectively 0.829, 0.860, and 0.880. Based on these findings, correlation between the two examiners and lumbar lordosis at different evaluated positions was very similar. This confirms the results of repeated measures ANOVA test. There was no difference between the two examiners and usage or not usage of two examiners simultaneously was the same. Correlation for examiners 1 and 2 and lumbar lordosis was 0.817 between flat surface and 10º wedge, 0.856 between flat surface and 15º wedge, and 0.871 between flat surface and 20º wedge. This shows that with increased angle, correlation increased as well. In analysis of thoracic kyphosis in order to evaluate the difference between variables and also between the two examiners, repeated measures ANOVA was used. The significance value for the variables was 0.000 and for the examiners it was 0.978. This demonstrates that there was difference regarding thoracic kyphosis between 4 positions studied, but not such difference existed between the examiners. Correlation for the examiner 1 and thoracic kyphosis at different standing positions between flat surface and 10º wedge, flat surface and 15º wedge, flat surface and 20º wedge were respectively 0.784, 0.824, 0.841. Correlation for the examiner 2 and thoracic kyphosis at different standing positions between flat surface and 10º wedge, flat surface and 15º wedge, flat surface and 20º wedge were respectively 0.833, 0.861, and 0.849. Based on these findings, correlation between the two examiners and thoracic kyphosis at different evaluated positions was very similar. Therefore, using correlation test it was revealed that no difference existed between the two examiners. Correlation for examiners 1 and 2 and thoracic kyphosis was 0.807 between flat surface and 10º wedge, 0.842 between flat surface and 15º wedge, and 0.844 between flat surface and 20º wedge. This shows that with increased angle, correlation increased as well.

In assessment made by examiner 1, lumbar lordosis increased by 1.8º (moving from flat surface to 10º wedge), 2.3º (moving from 10º to 15º wedge) and 2.5º (moving from wedge angled at 15º to 20º). In assessment made by examiner 2, lumbar lordosis increased by 2º (moving from flat surface to 10º wedge), 2.2º (moving from 10º to 15º wedge) and 2.2º (moving from wedge angled at 15º to 20º).

In assessment made by examiner 2, thoracic kyphosis increased by 0.8º (moving from flat surface to 10º wedge), 1.5º (moving from 10º to 15º wedge) and 1.4º (moving from wedge angled at 15º to 20º). In assessment made by examiner 2, thoracic kyphosis increased by 1º (moving from flat surface to 10º wedge), 1.3º (moving from 10º to 15º wedge) and 1.4º (moving from wedge angled at 15º to 20º).

The findings of this study show that comparison of lumbar lordosis and thoracic kyphosis at different positions (i.e., flat surface and on wedges angled at 10º, 15º, and 20º), there were significant differences (P< 0.0083), nut the difference was insignificant between the two examiners (P> 0.0083). Also, the results show that a positive correlation existed between the examiners and lumbar lordosis as well as thoracic kyphosis at four different positions studied. In other words, with increased foot pronation, lumbar lordosis and thoracic kyphosis increased as well. The results showed that with increased angle of the wedges and consequently increased foot pronation, lumbar lordosis and thoracic kyphosis increased. This is comparable to results reported by Khamis and Yizhar (2007), Pinto et al. (2008), and Tateuchi et al. (2011), (Khamis &Yizhar, 2007; Pinto, 2008; Tateuchi, Wada, & Ichihashi, 2011).

The most significant change between two measured positions occurred between flat surface and 10 degree wedge. did not report a significant relationship regarding mechanical relationship between posterior part of the foot and pelvis (Pinto, 2008). However, Khamis and Yizhar (2007), Pinto et al. (2008), and Tateuchi et al. (2011) reported significant increase between foot pronation increase and anterior tilt of the pelvis (Khamis & Yizhar, 2007; Pinto, 2008; Tateuchi, Wada, & Ichihashi, 2011).

Therefore, it can be concluded bilateral increase in foot pronation increase foot anteversion and anterior tilt of the pelvis. Possibly one of the reasons of not observing significant difference regarding thoracic kyphosis between a flat surface and 10° wedge is low quantity of the wedge’s angle and decreased effect of angle on upper parts of the trunk. Correlation between the two examiners regarding assessments made by them showed there was no significant difference between the two examiners regarding measurements made.

With increased the wedge’s angle and consequently foot pronation, thoracic kyphosis and lumbar lordosis increased, but the increased was less significant in thoracic kyphosis.

## 5. Conclusion

Considering this fact that foot is the most distal part of the lower extremity and acts as a support point through which the body maintains its balance, even minimal biomechanical changes at the support level can affect postural control strategies. If a change occurs in foot alignment, the pelvis changes its position in order to maintain the body’s center of gravity. In this study, with increased bilateral foot pronation towards 10, 15, and 20 degrees, lumbar lordosis increased to 1.9, 2.2, and 2.4 degrees, respectively. Likewise thoracic kyphosis increased to 0.9, 1.4, and 1.4 degrees, respectively. Each increase is a compensation that allows the body’s center of gravity to return to its stable position within the base of support’s span. With increased anterior tilt of the pelvis, lumbar vertebrae are deviated anteriorly and cause an increase in lumbar curvature. Therefore, the distance between gravity line from motional axis of the joint is increased which leads to increased extensor torque related to ideal normal position. Lumbar curvature increases and results in kyphosis to compensate increased lumbar curvature. In other words, increased kyphosis is compensation to increased lordosis and this increase is per se to compensate increased anterior tilt of the pelvis. Regarding the results of this study in determining the effect of increased foot pronation using wedges amongst healthy persons, possibly foot hyperpronation in individuals with flatfoot can cause increased lumbar lordosis and thoracic kyphosis.
